# Safety and Healthcare Resource Utilization in Patients Undergoing Left Atrial Appendage Closure—A Nationwide Analysis

**DOI:** 10.3390/jcm12144573

**Published:** 2023-07-09

**Authors:** Tharusan Thevathasan, Sêhnou Degbeon, Julia Paul, Darius-Konstantin Wendelburg, Lisa Füreder, Anna Leonie Gaul, Jan F. Scheitz, Gertraud Stadler, Andi Rroku, Sonia Lech, Pichit Buspavanich, Martin Huemer, Philipp Attanasio, Patrick Nagel, Markus Reinthaler, Ulf Landmesser, Carsten Skurk

**Affiliations:** 1 Department of Cardiology, Angiology and Intensive Care Medicine, Deutsches Herzzentrum der Charité (DHZC), Campus Benjamin Franklin, Hindenburgdamm 30, 12203 Berlin, Germany; 2 Berlin Institute of Health, Anna-Louisa-Karsch-Straße 2, 10178 Berlin, Germany; 3 Deutsches Zentrum für Herz-Kreislauf-Forschung e.V., Potsdamer Str. 58, 10785 Berlin, Germany; 4 Institute of Medical Informatics, Charité—Universitätsmedizin Berlin, Campus Mitte, Charitéplatz 1, 10117 Berlin, Germany; 5 Department of Neurology and Center for Stroke Research Berlin, Charité—Universitätsmedizin Berlin, Campus Benjamin Franklin, Hindenburgdamm 30, 12203 Berlin, Germany; 6 Research Unit Gender in Medicine, Charité—Universitätsmedizin Berlin, Campus Mitte, Charitéplatz 1, 10117 Berlin, Germany; 7 Institute for Medical Sociology and Rehabilitation Science, Charité—Universitätsmedizin Berlin, Campus Mitte, Charitéplatz 1, 10117 Berlin, Germany; 8 Department of Psychiatry and Neurosciences, Charité—Universitätsmedizin Berlin, Campus Mitte, Charitéplatz 1, 10117 Berlin, Germany; 9 Institute for Sexology and Sexual Medicine, Charité—Universitätsmedizin Berlin, Campus Mitte, Charitéplatz 1, 10117 Berlin, Germany

**Keywords:** left atrial appendage closure, atrial fibrillation, stroke, bleeding, sex difference, epidemiology

## Abstract

Percutaneous left atrial appendage closure (LAAC) has emerged as a non-pharmacological alternative for stroke prevention in patients with atrial fibrillation (AF) not suitable for anticoagulation therapy. Real-world data on peri-procedural outcomes are limited. The aim of this study was to analyze outcomes of peri-procedural safety and healthcare resource utilization in 11,240 adult patients undergoing LAAC in the United States between 2016 and 2019. Primary outcomes (safety) were in-hospital ischemic stroke or systemic embolism (SE), pericardial effusion (PE), major bleeding, device embolization and mortality. Secondary outcomes (resource utilization) were adverse discharge disposition, hospital length of stay (LOS) and costs. Logistic and Poisson regression models were used to analyze outcomes by adjusting for 10 confounders. SE decreased by 97% between 2016 and 2019 [95% Confidence Interval (CI) 0–0.24] (*p* = 0.003), while a trend to lower numbers of other peri-procedural complications was determined. In-hospital mortality (0.14%) remained stable. Hospital LOS decreased by 17% (0.78–0.87, *p* < 0.001) and adverse discharge rate by 41% (95% CI 0.41–0.86, *p* = 0.005) between 2016 and 2019, while hospital costs did not significantly change (*p* = 0.2). Female patients had a higher risk of PE (OR 2.86 [95% CI 2.41–6.39]) and SE (OR 5.0 [95% CI 1.28–43.6]) while multi-morbid patients had higher risks of major bleeding (*p* < 0.001) and mortality (*p* = 0.031), longer hospital LOS (*p* < 0.001) and increased treatment costs (*p* = 0.073). Significant differences in all outcomes were observed between male and female patients across US regions. In conclusion, LAAC has become a safer and more efficient procedure. Significant sex differences existed across US regions. Careful considerations should be taken when performing LAAC in female and comorbid patients.

## 1. Introduction

Atrial fibrillation (AF) is the most common cardiac arrhythmia worldwide and is estimated that it will reach a prevalence of 12.1 million individuals in the United States by 2030 [1,2]. Importantly, AF increases the stroke risk by four- to five-fold, with anticoagulation therapy being considered an effective therapy for stroke prevention based on patients’ individual stroke and bleeding risk (i.e., quantified by CHA_2_DS_2_-VASc and HAS-BLED scores) [3,4,5]. However, approximately 20–30% of patients with AF are considered to be poor candidates for long-term anticoagulation therapy due to increased bleeding risk (e.g., HAS-BLED score ≥ 3, underlying tumor, thrombocytopenia or prolonged triple therapy), drug intolerance, low adherence, or other contraindications [6,7].

The left atrial appendage (LAA) has been identified as the primary origin of emboli in more than 90% of embolic strokes in patients with non-valvular AF [8,9]. Hence, percutaneous left atrial appendage closure (LAAC) with an occluder device has emerged as a non-pharmacological alternative for stroke prevention [10]. The Watchman™ device (Boston Scientific, Marlborough, MA) was the first device to receive approval by the US Food and Drug Administration (FDA) in 2015 after two randomized controlled trials (RCTs) [11,12,13]. The Amulet™ device (St. Jude Medical, St. Paul, MN, USA) was approved in 2022 [14]. Several other LAAC devices are currently being tested in clinical trials [15,16], and novel indications for LAAC are being explored [17,18,19]. Long-term outcomes of the PREVAIL, PROTECT AF and PRAGUE-17 trials have shown that LAAC has similar efficacy to warfarin and new oral anticoagulants (i.e., prevention of stroke and systemic embolism (SE)) with lower non-procedural bleeding rates than, for which reason the prevention of complications and cost effectiveness of LAAC are important concerns [12,13,20].

LAAC entails a risk of peri-procedural complications, such as ischemic stroke, or pericardial effusion (PE), which may increase the burden on healthcare resource utilization due to increased hospital length of stay (LOS) and therapeutic costs. Recent RCTs and real-world registries have shown increased efficacy and improved safety of LAAC [14,21,22,23]. However, real-world data outside of these studies identifying risk factors of safety and resource utilization outcomes in patients undergoing LAAC are limited [22,24,25,26,27,28].

Therefore, in a large United States (US) national database comprising approximately one-fifth of all hospitals, we sought to investigate risk factors for in-hospital safety, defined as ischemic stroke, SE, PE, major bleeding, device embolization and in-hospital mortality, encompassing a time frame between FDA approval of the Watchman™ device (2015) and the onset of the COVID-19 pandemic (end of 2019). Secondary outcomes referred to healthcare resource utilization including hospital LOS, adverse discharge disposition and hospital costs.

## 2. Materials and Methods

### 2.1. Data Source

The study was performed following the Strengthening the Reporting of Observational Studies in Epidemiology (STROBE) checklist. Data were retrieved from the Healthcare Quality and Utilization Project National Inpatient Sample (HCUP NIS), which is an all-payer database of inpatient stays in the US that is made available by the Agency for Healthcare Research and Quality (AHRQ). Approval from the institutional review board was not mandated due to the public availability of the de-identified data. HCUP NIS data cover hospital-discharge-level data and represent an in-hospital sample of approximately 20% of US hospitals. The sample is considered to be geographically dispersed and representative of all inpatient admissions in the US [29].

The retrieved data included patient demographics (i.e., age, sex and ethnicity), primary payer status (i.e., public insurance by *Medicare and Medicaid*, private insurance, self-payment or other), patient diagnoses and procedures, as well as hospital characteristics (i.e., year of admission, hospital LOS, in-hospital mortality, hospital region, hospital location (urban or rural), teaching status, hospital size (small: 1–249 beds; medium: 250–449 beds; large: ≥450 beds), hospital costs and adverse discharge disposition). Patient diagnoses and procedures were retrieved from the International Classification of Diseases, Tenth Revision, Clinical Modification (ICD-10-CM), based on previously published ICD codes [26,30]. Deyo’s modification of the Charlson Comorbidity Index (CCI) was utilized to identify the level of patient comorbidities [31]. Additionally, the CHA_2_DS_2_-VASc-Score and a simplified form of the HAS-BLED score (criteria on International Normalized Ratio (INR) and antithrombotic medication not included) were calculated, based on previously published ICD codes [32,33,34]. Moreover, age groups were determined according to criteria of the CHA_2_DS_2_-VASc-Score: “young and middle-aged” (18–64 years), “senior” (65–74 years) and “gerontologic” patients (≥75 years). The patients’ cardiovascular risk burden was estimated on an ordinal scale by adding the number of cardiovascular risk factors for each patient. Risk factors included age (i.e., ≥55 years for males and ≥65 years for females) [35], hypertension, dyslipidemia, diabetes, peripheral vascular disease (PVD), as well as history of smoking, alcohol abuse or acute myocardial infarction [36].

### 2.2. Study Population

All hospitalized adult patients were included who were diagnosed with AF and received a percutaneous LAAC between 2016 and 2019, that is, the four years between FDA approval of the first LAAC device and the onset of the COVID-19 pandemic. AF and LAAC were selected based on the following ICD codes, as being previously published [26,30]: atrial fibrillation as I48.0, I48.1, I48.2, I48.91 as well as LAAC as 02L73 and subgroups.

### 2.3. Outcomes

The primary outcomes were defined as in-hospital peri-procedural complications and in-hospital mortality. In-hospital complications were defined based on previously published ICD codes [30,37]: stroke (i.e., ischemic stroke or transitory ischemic attack (TIA)); SE (i.e., embolization to non-cerebral organs); major bleeding in the intracranial, abdominal, respiratory, urinary, genital, orthopedic or ophthalmic system; PE and device embolization.

Outcomes on healthcare utilization were defined as secondary outcomes, including hospital LOS, adverse discharge disposition and hospital costs. Hospital LOS was defined as the number of days between hospital admission and discharge. Adverse discharge disposition was defined as discharge to another hospital or skilled nursing facility (SNF) [38]. Hospital costs were derived by converting hospital charges based on HCUP Cost-to-Charge ratios. Hospital charges included the amounts that hospitals billed for services, while hospital costs included the actual expenses for hospital services (including wages, supplies, and utilities).

### 2.4. Statistical Analysis

Data analyses were performed using R Core Team 2020, Version: 2023.06.0+421 (Vienna, Austria). Normality was assessed using Shapiro–Wilk analysis. Categorical and continuous variables were compared using Chi-square tests and Student’s *t*-tests (normally distributed variables) or Fisher’s exact test and Wilcoxon–Mann–Whitney U-test (non-normally distributed variables), respectively. Normally distributed continuous variables were expressed as mean (standard deviation, SD), non-normally distributed variables as median (interquartile range, IQR), and categorical variables as frequency (percentage). Results are presented as adjusted odds ratios (OR) or adjusted incidence rate ratios (IRR) with 95% confidence intervals (CI) and *p*-values. A two-tailed *p*-value of less than 0.05 was considered statistically significant.

Logistic regression was used to analyze the effects of risk factors on medical complications, in-hospital mortality and adverse discharge disposition. Poisson regression was utilized to test risk factors of hospital LOS and costs. Regression models were adjusted for age groups, sex, ethnicity, CCI, cardiovascular risk burden, renal failure, heart failure, hospital admission year, insurance status and hospital size.

### 2.5. Sensitivity Analyses

With an exploratory intent, the effects of risk factors on hospital LOS and adverse discharge disposition were analyzed in subgroups of low and high level of patient comorbidity (i.e., CCI ≤ 3 and >3), as well as patient sex (i.e., male and female). Each subgroup analysis was adjusted to the other nine confounders of the primary analyses. Additionally, analyses on hospital LOS were repeated after excluding patients who died during hospitalization to exclude bias derived from in-hospital mortality.

### 2.6. Geographic Analyses

To account for regional differences in healthcare, event rates for primary and secondary outcomes were examined for male and female patients across US regions and plotted on geographic maps.

## 3. Results

### 3.1. Patient Characteristics

**Figure 1:**
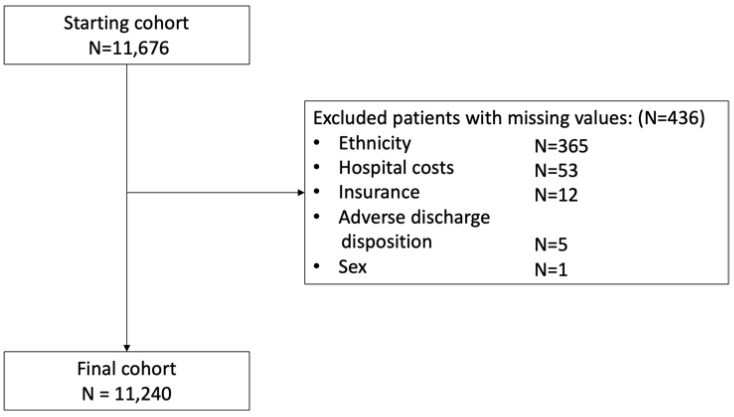
Patient flow diagram. This figure displays the number of patients with atrial fibrillation who underwent percutaneous left atrial appendage closure in the starting and final cohort after exclusion of patients with missing values.

Between 2016 and 2019, 11,676 patients with AF received a percutaneous LAAC. After excluding patients with missing values, 11,240 patients were included in the final study cohort (Table 1 and Figure 1). There was an approximately five-fold increase in LAAC cases from 2016 to 2019 (*p* < 0.001) (Table 1). In this study, cohort, 6534 patients (58.1%) were male, and 4706 patients (41.9%) were female. Most patients were of Caucasian ethnicity (9840 patients (87.5%)) and had a gerontologic age (6834 (60.8%)). The average CCI was 2 [1–3], the average CHA_2_DS_2_-VASc-Score was 4 [3–4] and the average simplified HAS-BLED score was 2 [2–3]. Patients had on average 4 [3–5] cardiovascular risk factors, mostly hypertension (9715 (86.4%)), dyslipidemia (6764 (60.2%)), PVD (4078 (36.3%)) and diabetes (3878 (34.5%)). Most LAAC cases (7531 (67.0%)) were performed in large hospitals and in publicly insured patients (10,100 (89.9%)).

### 3.2. Primary Outcome: Safety

**Figure 2:**
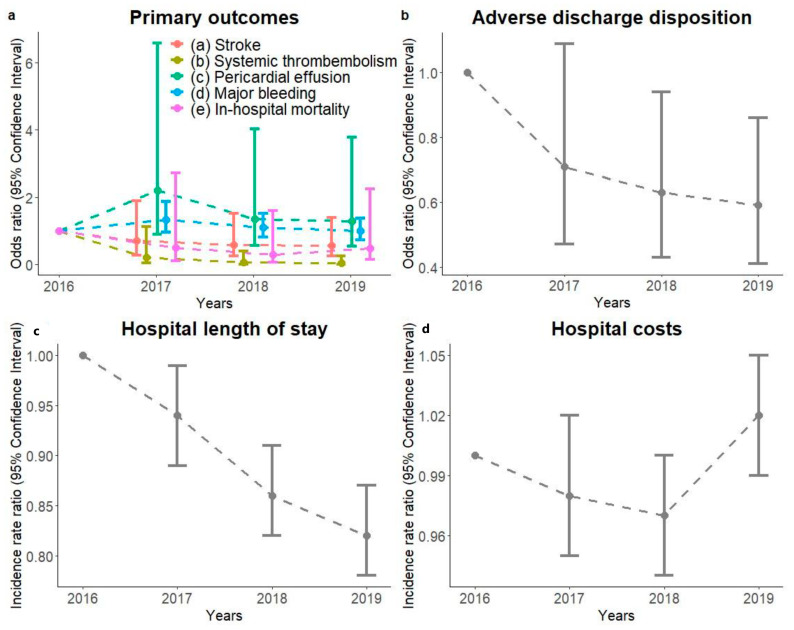
Peri-procedural outcomes stratified by year of left atrial appendage closure. Odds Ratios and Incidence Rate Ratio for primary (**a**) and secondary outcomes (**b**–**d**) are displayed for each year between 2016 and 2019.

**Peri-procedural complications.** Peri-procedural stroke occurred in 54 (0.5%) patients, with ischemic stroke occurring in 26 and a TIA in 28 patients. SE was observed in 9 (0.1%), PE in 85 (0.8%), major bleeding in 608 (5.4%) and device embolization in 1 (<0.01%) patient. Major bleeding occurred in the following organ systems (multiple counts per patient possible): abdominal *n* = 388; urinary *n* = 87; respiratory *n* = 35; intracranial *n* = 26; orthopedic *n*= 12; genital *n* = 9; ophthalmic *n* = 3 and other *n* = 14. While the peri-procedural complication rates showed a trend to lower numbers for all other complications between 2016 and 2019, a significant decrease by 97% [95% CI 0–0.24] was determined for SE (*p* = 0.004) (Figure 2, Table 2 and Table S1). Notably, female patients had a 286% [95% CI 2.41–6.39] higher risk of PE and a 500% [95% CI 1.28–43.6] higher risk of SE, while patients with a higher comorbidity level (CCI of 6) were characterized by a 198% [95% CI 1.78–4.92] higher risk of major bleeding.

**Peri-procedural mortality.** In total, 16 patients (0.1% of total study cohort) died after LAAC during their hospital stay (Table S2). These patients were mostly female (10 (62.5%)), elderly (81 years (75.5–85.25)) and had multiple comorbidities (CCI 3.5 (2.75–5)), including heart failure (10 patients (62.5%)) and renal failure (nine patients (56.3%)). The peri-procedural in-hospital mortality has been decreasing but did not change significantly between 2016 and 2019 (*p* = 0.3) (Figure 2, Table 2 and Table S1). Notably, female patients died more frequently than male patients after LAAC: 10 females (62.5%) compared to 6 males (37.5%); OR 2.57 [95% CI 0.92–7.80], *p* = 0.077. A higher patient comorbidity level was also associated with higher mortality risk (CCI of 6: OR 32.8 [95% CI 1.39–1,144.0], *p* = 0.031).

### 3.3. Secondary Outcomes: Healthcare Resource Utilization

**Adverse discharge disposition.** A total of 10,927 (97.2%) patients were successfully discharged home after LAAC. Adverse discharge after LAAC (i.e., to another hospital or a SNF) decreased by 41% [95% CI 0.41–0.86] from 3.9% (40 out of 1026) patients in 2016 to 2.5% (122 out of 4873) patients in 2019 (*p* = 0.005) (Table S3 and Figure 2). Patients with an adverse discharge disposition were mostly female (181 patients (57.8%)) (Table S3), who had an almost two-fold increased risk [95% CI 1.57–2.51] of adverse discharge disposition compared to male patients (*p* < 0.001). A higher patient comorbidity level was associated with a higher risk of adverse discharge disposition (CCI of 6: OR 5.42 [95% CI 2.63–11.0], *p* < 0.001 and heart failure: OR 1.32 [95% CI 1.02–1.72], *p* = 0.035).

**Hospital length of stay.** The average LAAC-related hospital LOS between 2016 and 2019 was 1 day [1–1], with 9311 (82.8%) patients staying in the hospital overnight. Hospital LOS decreased incrementally by 17% [95% CI 0.78–0.87] between 2016 and 2019 (*p* < 0.001) (Table S4 and Figure 2), indicating implementation of outpatient procedures. In particular, hospital LOS was prolonged in patients with multiple comorbidities (extended by 22% [95% CI 1.10–1.34] in patients with a CCI of 6, by 33% in patients with renal failure [95% CI 1.27–1.40] and by 25% in patients with heart failure [95% CI 1.21–1.30] (each *p* < 0.001). Results remained robust after excluding patients who died in the hospital (Table S4).

**Hospital costs.** The average costs for a LAAC-related hospital stay amounted to 24,719.7$ [18,992.6–31,193.0] per patient. Hospital costs remained stable between 2016 and 2019: IRR 1.02 [95% CI 0.99–1.05], *p* = 0.2 (Figure 2). Notably, LAAC performed in smaller hospitals was associated with 6% [95% CI 0.92–0.97] lower costs compared to LAAC in larger hospitals (*p* < 0.001). However, LAAC in patients with heart failure or renal failure was associated with 6% [95% CI 1.04–1.08] and 5% [95% CI 1.02–1.08] higher costs, respectively.

### 3.4. Subgroup Analyses

Primary analyses for hospital LOS (Table S5) and adverse discharge disposition (Table S6) were repeated in patient subgroups with low and high comorbidity levels, as well as in male and female patients. Hospital LOS and adverse discharge rate decreased between 2016 and 2019 across all subgroups. In general, female patients had a longer LOS and higher risk of adverse discharge disposition than male patients at each comorbidity level. Female patients with multiple comorbidities (i.e., CCI > 3) had a 30% [95% CI 1.22–1.38] longer hospital LOS than male patients, while female patients with fewer comorbidities (i.e., CCI ≤ 3) had an only 8% [95% 1.04–1.12] longer hospital LOS than male patients. Male patients had the most peculiar decrease in adverse discharge rate between 2016 and 2019, OR 0.45 [95% CI 0.27–0.77], while female patients with multiple comorbidities (i.e., CCI > 3) had a 103% [95% CI 1.35–2.81] higher risk of adverse discharge than male patients. Importantly, the characteristics of male and female patients who died after LAAC differed: compared to dead male patients, dead female patients were older (81.4 years vs. 75.0 years), had a higher CHA_2_DS_2_VASc score (5.0 [4.0–5.75] vs. 3.0 [3.0–3.75]) and a higher rate of heart failure (70% vs. 50%) (Table S7).

### 3.5. Geographic Analyses

**Figure 3:**
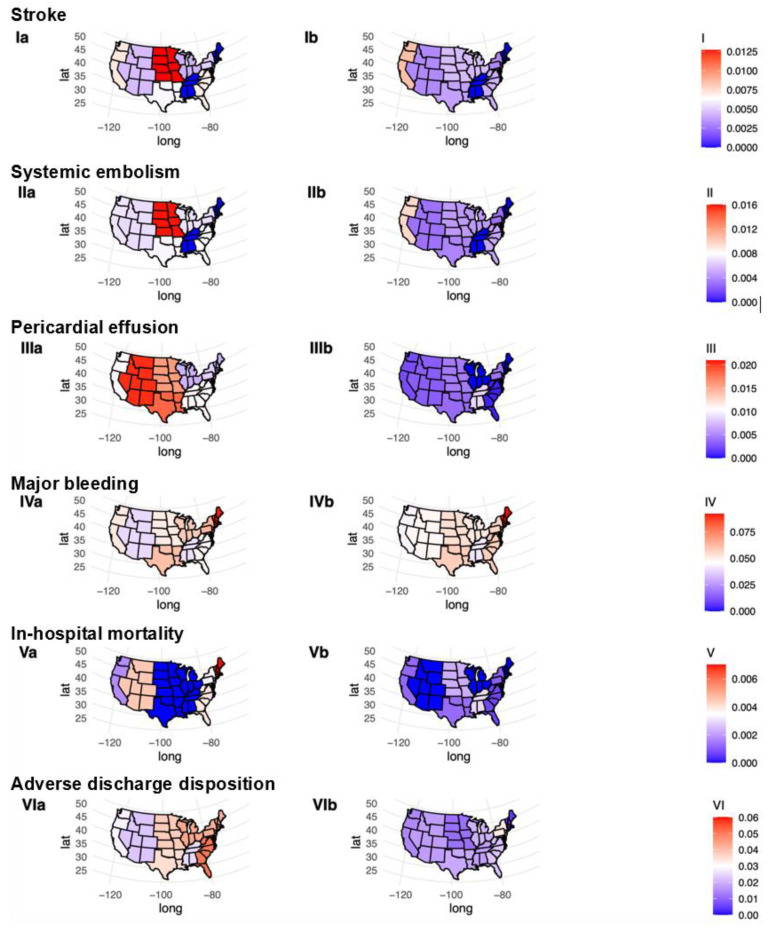
Peri-procedural in-hospital event rates stratified by female and male patients undergoing left atrial appendage closure across US regions. This figure displays the event rates for peri-procedural outcomes (**I**–**VI**) in female (**a**) and male (**b**) patients. Shades of blue color indicate lower event rates, while shades of red color indicate higher event rates.

Strong variations in peri-procedural complications, mortality and adverse discharge disposition existed between male and female patients across the US (Figure 3). Male patients had a more homogenous distribution of event rates than female patients. In female patients, stroke and SE rates were particularly high in northern US regions, while PE rates were higher in western US regions. Major bleeding rates were more evenly distributed in both sexes across the US.

## 4. Discussion

In this large nationwide study, we showed that LAAC became a safer and more efficient procedure between 2016 and 2019, with numerically fewer complication rates, significantly attenuated rates of SE, a relatively low overall in-hospital mortality rate, shorter hospitalizations, and fewer adverse discharge rates. However, hospital costs did not change significantly during the study period. Strong sex differences in peri-procedural outcomes existed across US regions. Specifically, female patients and patients with multiple comorbidities showed worse outcomes.

The first RCTs on LAAC were characterized by relatively high rates of peri-procedural complications within 7 days after the procedure [11,12]. However, improved implantation techniques, better peri-procedural imaging techniques of the LAA and the transseptal puncture site, novel device developments, and improved vascular access techniques (e.g., ultrasound-guided puncture with micropuncture needle) and vascular closure techniques (e.g., figure-of-eight suture or suture-mediated preclosure), as well as specific delivery sheaths, have led to peri-procedural complication rates of less than 3% in most recent RCTs [14,21,39]. These improvements have also been shown in recent real-world registries [22,23]. In this study, we corroborate these findings in a relatively large study cohort of approximately one-fifth of all hospitalization in the US: Complication rates have numerically decreased since 2016, in particular with a significant reduction in SE rates.

Transseptal puncture or local trauma to the LAA may result in PE and cardiac tamponade, which have been reported to be the main drivers of safety outcomes after LAAC [11,12]. Approximately 90% of PE occur within the first 24 h after LAAC, with rates of 1–2% [8,14,22,40,41], which are similar to the finding of 0.8% in this study. Advancements in operator experience, pre-procedural imaging, transesophageal (TEE) or intracardiac (ICE) echocardiographic guidance, adequate selection of transseptal puncture sites and advanced delivery systems reduce trauma to the LAA [42]. Notably, female patients who underwent LAAC had an almost three-fold higher risk of PE than male patients in this study, which is similar to very recently published findings in the Amulet IDE Trial [43]. Female sex was recently identified as an independent risk factor of PE given sex-specific differences in structural and physiological characteristics of the atrium [44,45,46,47,48]. In this study, cohort, female patients undergoing LAAC had a different risk profile (e.g., age and comorbidity level) from male patients, as reported previously [44]. However, this study was not designed to explain the underlying mechanisms for the observed sex differences. Several plausible explanations exist, such as sex-based anatomical differences (left atrial size or LAA morphology) between women and men may increase the risk of medical complications in women [49]. Additionally, it has been proposed that female AF patients may have more prominent atrial remodeling and fibrosis with reduced LAA elasticity, further complicating LAAC [50]. Lastly, women were less likely to be prescribed oral anticoagulants or single antiplatelet therapy [44]. Further sex-specific clinical studies are required to explain the underlying mechanisms for sex-based differences after LAAC. Therefore, further peri-procedural caution is warranted for female patients undergoing LAAC, similar to other procedures such as transfemoral transcatheter aortic valve replacement or myocardial revascularization [44,51,52,53].

The purpose of LAAC is stroke prevention, wherefore mitigation of peri-procedural stroke is vital. In this study, patients with a high stroke risk were included (i.e., CHA_2_DS_2_-VASc score 4 [3,4]). Stroke occurred in 0.5% and SE in 0.1% after LAAC during hospitalization, which was lower than the reported peri-procedural stroke rates of 1–3% reported across a range of studies and device types [11,14,54,55]. A meta-analysis of the randomized trials PROTECT AF, PREVAIL and PRAGUE-17 showed non-inferiority of LAAC in prevention of stroke and SE with fewer non-procedural bleeding rates compared to medical therapy with anticoagulants [56]. Peri-procedural thromboembolic complications may originate from the left atrium, the LAA or from the implantation procedure itself. Hence, procedural stroke might be prevented by careful pre-procedural echocardiographic screening for thrombus, judicious flushing of delivery systems (preventing air embolus), sufficient procedural anticoagulation and careful pre-procedural planning. Similar to PE, female patients had a higher risk of SE compared to male patients in this study, as confirmed in previous registries [57,58].

Major bleeding was the most frequent complication, being experienced by 5.4% of the patients in this study cohort, which consisted of patients with an intermediate bleeding risk (i.e., simplified HAS-BLED score 2 [2,3]). The HAS-BLED score in this study cohort was comparable to that in the EWOLUTION registry [59]. Although the HAS-BLED score was not prospectively captured in the Watchman™ studies, many components of the HAS-BLED score were captured as part of routine data collection. In those studies, a conservative bleeding score was determined using the available case report form data and points were assigned as per the HAS-BLED score. In this study, the international normalized ratio and medication were not part of the simplified HAS-BLED score. Other studies have reported major bleeding rates of approximately 2–10% [23,59,60]. In particular, patients with multiple comorbidities had a higher risk of experiencing major bleeding in this study. The importance of the patients’ comorbidity level on bleeding risk is mirrored in the HAS-BLED score, which includes comorbidities such as hypertension, chronic renal or liver malfunction, and history of bleeding.

Overall peri-procedural mortality in this study cohort was low, at 16 patients, which is similar to other studies [30]. Patients who died in the hospital were older (75% of deceased patients were ≥75 years old), mostly female, and had more comorbidities. A very recently published multicenter study with 807 patients showed that older age, low body mass index, and patient comorbidities were predictors of one-year mortality [24]. Although LAAC might be appealing for frail and comorbid patients who have a high bleeding risk, the same patient group is particularly vulnerable to peri-procedural mortality and complications after LAAC. Therefore, adequate patient selection is important in real-world practice [24,61].

As LAAC is becoming a widespread procedure, improvements in healthcare resource utilization and cost effectiveness are necessary. In this study, LAAC was found to be an efficient procedure: Hospital LOS and adverse discharge rates decreased by 17% and 41% between 2016 and 2019, respectively. These strong trends might be a result of progress in operator experience and improved LAAC techniques with adequate peri-procedural preparation and monitoring. Hospital LOS was 1 day [1–1] on average, which is in alignment with international standard practice of post-procedural overnight monitoring and hospital discharge on the following day [28,62,63,64], in contrast to the hospitalizations of 4.6 days in the era prior to FDA approval of the Watchman device in 2015 [65]. In particular, LOS appears to be longer in female patients across multiple analyses, corroborating increased peri-procedural complication rates. Therefore, careful consideration should be taken when performing LAAC in women. Despite the short hospital LOS in this study, hospital costs remained stagnant over the study period. In this regard, same-day discharge strategies after elective LAAC have been proposed, which may result in improved patient satisfaction and lower hospital costs [63].

Importantly, the main and subgroup analyses confirmed that patients with multiple comorbidities posed a stronger burden on healthcare resources, with prolonged hospitalizations and increased adverse discharge rates due to an increased risk of peri-procedural complications, potentially resulting in significantly increased hospital costs. Therefore, careful peri-procedural considerations should be taken when performing LAAC in patients with multiple comorbidities to improve patient-level and hospital-level outcomes, as was also concluded in another recent study [24]. Peri-procedural complications are a major trigger for prolonged hospitalization, wherefore strategies for short hospitalizations should be applied to low-risk patients only [62].

Notably, strong variations in LAAC-related healthcare resource utilization have been reported between countries [62]. In this study, strong variations in peri-procedural complications, mortality and adverse discharge disposition existed between male and female patients across the United States. These findings suggest inter-regional differences in provider experience, hospital case volumes and standards of care. Therefore, standardized national approaches should be considered to provide homogenous quality of care across hospital regions.

This study should be interpreted in the context of certain limitations: The HCUP NIS data did not include information on laboratory, medication (e.g., antithrombotic or anticoagulation therapy) or imaging (e.g., peri-device leak) reports. The majority of patient data represented publicly insured patients similar to other LAAC studies [66]. Given the retrospective design, selection bias or confounding by indication could not be ruled out, although the analyses were adjusted for multiple a priori defined patient- and hospital-specific confounders. State-level analyses were not performed, as the NIS is not considered to be representative of each individual US state; analyses on US hospital regions (comprised of multiple states) were performed instead. Additionally, the analyses were restricted to short-term in-hospital outcomes following LAAC. Nonetheless, this study made it possible to analyze risk factors on safety and healthcare resource utilization outcomes in a large nationwide cohort of patients undergoing LAAC in a real-world setting and the results were based on “as-treated analyses”.

## 5. Conclusions

In conclusion, LAAC has become a safer and more efficient procedure with fewer complications and lower adverse discharge rates. Strong sex differences in peri-procedural outcomes existed between male and female patients across US regions. Importantly, female patients and patients with multiple comorbidities had worse outcomes, and therefore caution is warranted in these patients. Further studies are required to investigate differences in long-term safety and hospital resource utilization after LAAC.

## Figures and Tables

**Table 1:** Characteristics of 11,240 patients with atrial fibrillation receiving left atrial appendage closure between 2016 and 2019.

Characteristics	Value
Year of LAAC - 2016 - 2017 - 2018 - 2019	1026 (9.1) 2054 (18.3) 3287 (29.2) 4873 (43.4)
- Age (years) - Young and middle-aged (18–64 years) - Senior (65–74 years) - Gerontologic (≥75 years)	77 [71–82] 834 (7.4) 3572 (31.8) 6834 (60.8)
Sex - Female - Male	4706 (41.9) 6534 (58.1)
Ethnicity - Caucasian - Latin-American - Afro-American - Asian - Native Americans - Other	9840 (87.5) 557 (5.0) 460(4.1) 157 (1.4) 38 (0.3) 188 (1.7)
Hospital size - Large (≥450 beds) - Medium (250–449 beds) - Small (1–249 beds)	7531 (67.0) 2578 (22.9) 1131 (10.1)
Insurance - Public - Private - Self-payment - Other	10,100 (89.9) 910 (8.1) 52 (0.5) 178 (1.6)
CCI - CCI > 3 - CCI ≤ 3	2.00 [1.0–3.0] 2511 (22.3) 8729 (77.7)
Heart failure	3835 (34.1)
Renal failure	2730 (24.3)
Cardiovascular risk burden	4 [3–5]
Cardiovascular risk factors - Male >55 years - Female >65 years - Hypertension - Dyslipidemia - Current or past smoker - History of myocardial infarction - Alcohol abuse - Peripheral vascular disease - Diabetes	6427 (57.2) 4435 (39.5) 9715 (86.4) 6764 (60.2) 4078 (36.3) 1402 (12.5) 125 (1.1) 4078 (36.3) 3878 (34.5)
Hospital region - New England - Middle Atlantic - East North Central - West North Central - South Atlantic - East South Central - West South Central - Mountain - Pacific	350 (3.1) 1551 (13.8) 1606 (14.3) 759 (6.8) 2444 (21.7) 617 (5.5%) 1443 (12.8) 1081 (9.6) 1389 (12.4)
CHA_2_DS_2_-VASc score	4 [3–4]
Simplified HAS-BLED score	2 [2–3]
Hospital location and teaching status - Rural - Urban and non-teaching - Urban and teaching	212 (1.9) 1057 (9.4) 9971 (88.7)

Values are displayed as frequency (percent), mean (standard deviation) or median [interquartile range]. Charlson Comorbidity Index (CCI); left atrial appendage closure (LAAC).

**Table 2:** Results for primary outcomes (safety outcomes).

Characteristics	Stroke (*n* = 54)	Systemic Embolism (*n* = 9)	Pericardial Effusion (*n* = 85)	Major Bleeding (*n* = 608)	In-Hospital Mortality (*n* = 16)
Year of LAAC					
- 2016	1	1	1	1	1
- 2017	0.70 (0.27–1.89)	0.21 (0.03–1.11)	2.19 (0.89–6.59)	1.32 (0.95–1.86)	0.49 (0.09–2.71)
- 2018	0.58 (0.24–1.51)	0.05 (0.00–0.38)	1.34 (0.55–4.02)	1.09 (0.80–1.52)	0.29 (0.05–1.59)
- 2019	0.56 (0.25–1.39)	0.03 (0.00–0.24)	1.28 (0.54–3.78)	0.99 (0.73–1.36)	0.47 (0.13–2.24)
Age groups					
- Gerontologic	1	1	1	1	1
- Senior	0.74 (0.39–1.35)	0.31 (0.02–1.80)	0.76 (0.46–1.24)	0.93 (0.77–1.12)	0.52 (0.12–1.66)
- Young and middle-aged	0.37 (0.06–1.33)	1.21 (0.03–15.2)	0.52 (0.15–1.40)	0.86 (0.60–1.20)	0.73 (0.04–4.09)
Sex - Male - Female	1 1.36 (0.78–2.37)	1 6.00 (1.28–43.6)	1 3.86 (2.41–6.39)	1 0.99 (0.83–1.17)	1 2.57 (0.92–7.80)
Insurance					
- Public	1	1	1	1	1
- Private	1.03 (0.28-2.84)	1.91 (0.06-22.5)	1.81 (0.79-3.67)	1.26 (0.92-1.70)	-
- Self-payment	-	-	-	0.36 (0.02-1.65)	-
- Other	-	-	1.26 (0.07-5.94)	0.92 (0.43-1.73)	-
Ethnicity					
- Caucasian	1	1	1	1	1
- Latin-American	0.70 (0.11–2.30)	-	0.66 (0.16–1.79)	0.85 (0.55–1.25)	-
- Afro-American	0.39 (0.02–1.83)	-	0.96(0.29–2.36)	1.34 (0.92–1.91)	-
- Asian	0.97 (0.05–5.20)	-	0.82 (0.05–3.85)	1.19 (0.58–2.17)	3.96 (0.21–21.7)
- Native Americans	-	-	-	2.07 (0.61–5.26)	
- Other	0.91 (0.05–4.34)	-	2.82 (0.84–7.03)	1.19 (0.62–2.06)	3.32 (0.18–18.0)
Hospital size					
- Large	1	1	1	1	1
- Medium	1.13 (0.57–2.11)	1.69 (0.33–7.18)	1.02 (0.59–1.70)	1.00 (0.81–1.21)	0.44 (0.07–1.63)
- Small	0.85 (0.29–2.03)	-	1.42 (0.70–2.64)	0.86 (0.63–1.14)	0.53 (0.03–2,75)
CCI					
- 0	1	1	1	1	1
- 1	-	0.75 (0.05–19.3)	0.84 (0.39–1.79)	1.05 (0.79–1.38)	1.45 (0.05–38.4)
- 2	-	0.86 (0.05–25.2)	0.99 (0.44–2.21)	1.22 (0.91–1.65)	4.66 (0.38–109)
- 3	-	0.20 (0.00–12.2)	0.81 (0.29–2.16)	1.58 (1.11–2.23)	15.9 (1.45–376)
- 4	-	-	0.59 (0.18–1.83)	1.86 (1.25–2.76)	18.6 (1.37–482)
- 5	-	0.15(0.00–18.8)	0.55 (0.14–1.99)	1.82 (1.12–2.91)	19.2 (0.97–591)
- 6	-	-	0.82 (0.20–3.10)	2.98 (1.78–4.92)	32.8(1.39–1,144)
- 7	-	-	0.63 (0.08–3.29)	3.18 (1.68–5.83)	36.0(0.82–1,498)
- 8	-	-	4.58 (0.79–20.8)	1.46 (0.34–4.28)	-
- 9	-	-	-	3.69 (0.85–11.2)	-
- 10	-	-	-	21.2 (4.01–101)	-
- 11	-	1035 (-)	-	9.87 (1.36–48.5)	-
- 12	-	0.03 (-)	-	-	-
- 13	-	-	-	10.1 (0.49–82.5)	-
- 14	-	-	-	-	-
- 15	-	-	-	-	-
- 16	-	-	-	-	-
- 17	-	1.23 (-)	-	-	-
Heart failure	0.76 (0.42–1.35)	6.30 (1.07–54.0)	1.64 (0.97–2.80)	1.02 (0.84–1.25)	1.31 (0.43–4.47)
Renal failure	0.31 (0.14–0.66)	5.06 (0.28–66.9)	2.23 (1.10–4.52)	0.83 (0.64–1.09)	1.17 (0.32–4.76)
Cardiovascular					
risk burden					
- 0	1	1	1	1	1
- 1	0.16 (0.01–3.80)	-	0.32 (0.04–6.68)	1.02 (0.28–6.53)	-
- 2	0.04 (0.00–0.89)	-	0.16 (0.02–3.37)	0.85 (0.24–5.36)	-
- 3	0.04 (0.00–0.95)	-	0.16 (0.02–3.38)	0.83 (0.24–5.27)	-
- 4	0.03 (0.00–0.83)	-	0.27 (0.03–5.51)	0.77 (0.22–4.87)	-
- 5	0.02 (0.00–0.57)	3.83 (-)	0.25 (0.03–5.29)	0.68 (0.19–4.87)	-
- 6	0.01 (0.00–0.32)	-	0.24 (0.03–5.32)	0.60 (0.16–3.84)	-
- 7	0.04 (0.00–1.10)	0.90 (-)	0.00 (0–429)	0.45 (0.11–3.11)	-
- 8	-	163 (-)	-	-	-

Values are displayed as odds ratios and 95% confidence intervals. Device embolization was not included as an outcome due to few events (*n* = 1). Charlson Comorbidity Index (CCI); left atrial appendage closure (LAAC).

## Data Availability

Data were retrieved from the Healthcare Quality and Utilization Project National Inpatient Sample (HCUP NIS) which is an all-payer database of inpatient stays in the US that is made available by the Agency for Healthcare Research and Quality (AHRQ) at https://hcup-us.ahrq.gov/nisoverview.jsp accessed on 1 March 2022.

## Supplementary Materials

The following supporting information can be downloaded at: https://www.mdpi.com/article/10.3390/jcm12144573/s1, Table S1. Frequency of peri-procedural complications and mortality in patients receiving left atrial appendage closure between 2016 and 2019; Table S2. Characteristics of patients with atrial fibrillation receiving left atrial appendage closure between 2016 and 2019 stratified by survival status; Table S3. Characteristics of patients with atrial fibrillation receiving left atrial appendage closure between 2016 and 2019 stratified by adverse discharge disposition; Table S4. Results for hospital length of stay in patients without in-hospital mortality (*n* = 11,224); Table S5. Results for hospital length of stay in patient subgroups stratified by patient comorbidity and sex; Table S6. Results for adverse discharge disposition in patient subgroups stratified by patient comorbidity and sex. Table S7. Characteristics of dead patients stratified by sex.
