# Supplementary material for: Safety and Healthcare Resource Utilization in Patients Undergoing Left Atrial Appendage Closure—A Nationwide Analysis
Source: J Clin Med. 2023 Jul 9;12(14):4573. doi: 10.3390/jcm12144573 (PMC10380523; doi:10.3390/jcm12144573)
Supplement: Supplementary file 1 [file jcm-12-04573-s001.zip › jcm-2447302-supplementary.pdf]

Supplementary Material

Safety and Healthcare Resource Utilization in Patients Undergoing Left-Atrial Appendage Closure - A Nationwide Analysis.

Tharusan Thevathasan MD[1,2,3,4†*], Sêhnou Degbeon cand. med.[1†], Julia Paul cand. med.[1], Darius-Konstantin Wendelburg cand. med.[1], Lisa Füreder MD[1], Anna Leonie Gaul cand. med.[1], Jan F. Scheitz MD[2,3,5], Gertraud Stadler MD[6], Andi Rroku MD[1,3], Sonia Lech MSc[7,8], Pichit Buspavanich MD[6,9], Martin Huemer MD[1], Philipp Attanasio MD[1], Patrick Nagel MD[1], Markus Reinthaler MD[1,3], Ulf Landmesser MD[1,2,3], Carsten Skurk MD[1,3*]

*** Correspondence:**

Carsten Skurk MD and Tharusan Thevathasan MD
Department of Cardiology, Angiology and Intensive Care Medicine,
Deutsches Herzzentrum der Charité (Campus Benjamin Franklin),
Hindenburgdamm 30, 12203 Berlin, Germany
Phone: +49 30 450 513 702; Fax: +49 30 450 513 999;
Email: carsten.skurk@dhzc-charite.de and tharusan.thevathasan@dhzc-charite.de

Table S1. Frequency of peri-procedural complications and mortality in patients receiving left atrial appendage closure between 2016 and 2019

Table S2. Characteristics of patients with atrial fibrillation receiving left atrial appendage closure between 2016 and 2019 stratified by survival status

Table S3. Characteristics of patients with atrial fibrillation receiving left atrial appendage closure between 2016 and 2019 stratified by adverse discharge disposition

Table S4. Results for hospital length of stay in patients without in-hospital mortality (N=11,224)

Table S5. Results for hospital length of stay in patient subgroups stratified by patient comorbidity and sex

Table S6. Results for adverse discharge disposition in patient subgroups stratified by patient comorbidity and sex

Table S7. Characteristics of dead patients stratified by sex

Table S1. Frequency of peri-procedural complications and mortality in patients receiving left atrial appendage closure between 2016 and 2019

**Year**	**2016 (N=1,026)**	**2017 (N=2,054)**	**2018 (N=3,287)**	**2019 (N=4,873)**
**Stroke**	8 (0.8%)	11 (0.5%)	14 (0.4%)	21 (0.4%)
**Systemic embolism**	5 (0.5%)	2 (0.1%)	1 (0.0%)	1 (0.0%)
**Pericardial effusion**	5 (0.5%)	23 (1.1%)	24 (0.7%)	33 (0.7%)
**Major bleeding**	51 (5.0%)	133 (6.5%)	181 (5.5%)	243 (5.0%)
**In-hospital mortality**	3 (0.3%)	3 (0.1%)	3 (0.1%)	7 (0.1%)

Values are displayed as frequency (percent).

Table S2. Characteristics of patients with atrial fibrillation receiving left atrial appendage closure between 2016 and 2019 stratified by survival status

Characteristics	Patients who died during hospitalization (N=16)	Patient who survived (N=11,224)
**Year of LAAC**		
- 2016	3 (18.8)	1,023 (9.1)
- 2017	3 (18.8)	2,051 (18.3)
- 2018	3 (18.8)	3,284 (29.3)
- 2019	7 (43.8)	4,866 (43.4)
**Age (years)**	81 [75.5-85.25]	77 [71.0-76.05]
- Young and middle-aged (18-64 years)	1 (6.2)	833 (7.4)
- Senior (65-74 years)	3 (18.8)	3,569 (31.8)
- Gerontologic (≥75 years)	12 (75)	6,822 (60.8)
**Sex**		
- Female	10 (62.5)	4,696 (41.8)
- Male	6 (37.5)	6,528 (58.2)
**Ethnicity**		
- Caucasian	14 (87.5)	9,826 (87.5)
- Latin- American	0 (0)	557 (5.0)
- Afro-American	0 (0)	460 (4.1)
- Asian	1 (6.3)	156 (1.4)
- Native Americans	0 (0)	38 (0.3)
- Other	1 (6.3)	187 (1.7)
**Hospital size**		
- Large (≥250 beds)	13 (81.3)	7,518 (67.0)
- Medium (250-449 beds)	2 (12.5)	2,576 (23.0)
- Small (1-249 beds)	1 (6.3)	1,130 (10.1)
**Insurance**		
- Public	16 (100)	10,084 (89.8)
- Private	0	910 (8.1)
- Self-payment	0	52 (0.5)
- Other	0	178 (1.6)
**CCI**	3.5 [2.75-5.0]	2 [1.0-3.0]
- CCI >3	8 (50)	2,503 (22.3)
- CCI ≤3	8 (50)	8,721 (77.7)
**Heart failure**	10 (62.5)	3,825 (34.1)
**Renal failure**	9 (56.3)	2,721 (24.2)
**Cardiovascular risk burden**	3 [2.0-4.25]	4 [3.0-5.0]

**Cardiovascular risk factors**		
- Male >55 years	6 (37.5)	6,421 (57.2)
- Female >65 years	9 (56.3)	4,426 (39.4)
- Hypertension	12(75.0)	9,703 (86.4)
- Dyslipidemia	6 (37.5)	6,758 (60.2)
- Current or past smoker	4 (25.0)	4,074 (36.3)
- History of myocardial infarction	2 (12.5)	1,400 (12.5)
- Alcohol abuse	0	125 (1.1)
- Peripher vascular disease	4 (25.0)	4,074 (36.3)
- Diabetes	7 (43.8)	3,871 (34.5)
**Hospital region**		
- New England	1 (6.3)	349 (3.1)
- Middle Atlantic	3 (18.8)	1,548 (13.8)
- East North Central	0	1,606 (14.3)
- West North Central	1 (6.3)	758 (6.8)
- South Atlantic	5 (31.1)	2,439 (21.7)
- East South Central	1 (6.3)	616 (5.5)
- West South Central	1 (6.3)	1,442 (12.8)
- Mountain	2 (12.5)	1,079 (9.6)
- Pacific	2 (12.5)	1,387 (12.4)
**CHADsVASc score**	4 [3-5]	4 [3-4]
**Simplified HAS-BLED score**	3 [1.75-3]	2 [2-3]
**Hospital location and teaching status**		
- Rural	1 (6.3)	211 (1.9)
- Urban and non-teaching	1 (6.3)	1,056 (9.4)
- Urban and teaching	14 (87.5)	9,957 (88.7)

Values are displayed as frequency (percent), mean (standard deviation) or median [interquartile range]. Charlson Comorbidity Index (CCI); left atrial appendage closure (LAAC).

Table S3. Characteristics of patients with atrial fibrillation receiving left atrial appendage closure between 2016 and 2019 stratified by adverse discharge disposition

**Characteristics**	**Patient with adverse discharge disposition (N=313)**	**Patient without adverse discharge disposition (N=10,927)**
**Year of LAAC**		
- 2016	40 (12.8)	986 (9.0)
- 2017	61(19.5)	1,993 (18.2)
- 2018	90 (28.8)	3,197 (29.3)
- 2019	122 (39.0)	4,751 (43.5)
**Age (years)**	78 [73.0-82.0]	77 [71.0-82.0]
- Young and middle-aged (18-64 years)	19 (6.1)	815 (7.5)
- Senior (65-74 years)	85 (27.2)	3,487 (31.9)
- Gerontologic (≥75 years)	209 (66.8)	6,625 (60.6)
**Sex**		
- Female	181 (57.8)	4,525 (41.4)
- Male	132 (42.2)	6,402 (58.6)
**Ethnicity**		
- Caucasian	272 (86.9)	9,568 (87.6)
- Latin- American	19 (6.1)	538 (4.9)
- Afro-American	12 (3.8)	448 (4.1)
- Asian	5 (1.6)	152 (1.4)
- Native Americans	0 (0)	38 (0.3)
- Other	5 (1.6)	183 (1.7)
**Hospital size**		
- Large (≥250 beds)	206 (65.8)	7,325 (67.0)
- Medium (250-449 beds)	75 (24.0)	2,503 (22.9)
- Small (1-249 beds)	32 (10.2)	1,099 (10.1)
**Insurance**		
- Public	287 (91.7)	9,813 (89.8)
- Private	21 (6.7)	889 (8.1)
- Self-payment	2 (0.6)	50 (0.5)
- Other	3 (1.0)	175 (1.6)
**CCI**	3.0 [1.0-4.0]	2.0 [1.0-3.0]
- CCI >3	106 (33.9)	2,405 (22.0)
- CCI ≤3	207 (66.1)	8,522 (78.0)
**Heart failure**	158 (50.5)	3,677 (33.7)
**Renal failure**	109 (34.8)	2,621 (24.0)
**Cardiovascular risk burden**	3 [2.0-5.0]	4 [4.0-5.0]

**Cardiovascular risk factors**		
- Male >55 years	132 (42.2)	6,295 (57.6)
- Female >65 years	170 (54.3)	4,265 (39.0)
- Hypertension	269 (85.9)	9,446 (86.4)
- Dyslipidemia	172 (55.0)	6,592 (60.3)
- Current or past smoker	100 (31.9)	3,978 (36.4)
- Prior myocardial infarction	47 (15.0)	1,355 (12.4)
- Alcohol abuse	10 (3.2)	115 (1.1)
- Peripher vascular disease	100 (31.9)	3,978 (36.4)
- Diabetes	120 (38.3)	3,758 (34,4)
**Hospital region**		
- New England	7 (2.2)	343 (3.1)
- Middle Atlantic	57 (18.2)	1,494 (13.7)
- East North Central	49 (15.7)	1,557 (14.2)
- West North Central	17 (5.4)	742 (6.8)
- South Atlantic	83 (26.5)	2,361 (21.6)
- East South Central	13 (4.2)	604 (5.5)
- West South Central	38 (12.1)	1,405 (12.9)
- Mountain	21 (6.7)	1,060 (9.7)
- Pacific		
	28 (8.9)	1,361 (12.5)
**CHADsVASc score**	4 [3.0,5.0]	4 [3.0,4.0]
**Simplified HAS-BLED score**	2 [2.0,3.0]	2 [2.0,2.0]
**Hospital location and teaching status**		
- Rural	7 (2.2)	205 (1.9)
- Urban and non-teaching	34 (10.9)	1,023 (9.4)
- Urban and teaching	272 (86.9)	9,699 (88.8)

Values are displayed as frequency (percent), mean (standard deviation) or median [interquartile range]. Charlson Comorbidity Index (CCI); left atrial appendage closure (LAAC).

Table S4. Results for hospital length of stay in patients without in-hospital mortality (N=11,224)

Characteristics	Hospital length of stay
**Year of LAAC**	
- 2016	1
- 2017	0.94 (0.89-1)
- 2018	0.86 (0.82-0.91)
- 2019	0.83 (0.78-0.87)
**Age groups**	
- Gerontologic	1
- Senior	1.01 (0.97-1.04)
- Young and middle-aged	1.18 (1.11-1.25)
**Sex**	
- Male	1
- Female	1.13 (1.09-1.16)
**Insurance status**	
- Public	1
- Private	1.19 (1.13-1.26)
- Self-payment	1.22 (0.99-1.49)
- Other	0.93 (0.81-1.05)
**Ethnicity**	
- Caucasian	1
- Latin- American	1.12 (1.05-1.20)
- Afro-American	1.06 (0.98-1.14)
- Asian	1.11 (0.98-1.25)
- Native Americans	0.98 (0.75-1.25)
- Other	1.18 (1.05-1.31)
**Hospital size**	
- Large	1
- Medium	0.99 (0.95-1.02)
- Small	0.93 (0.88-0.98)
**CCI**	
- 0	1
- 1	1.07 (1.02-1.13)
- 2	1.13 (1.07-1.20)
- 3	1.17 (1.09-1.25)
- 4	1.05 (0.97-1.13)
- 5	1.04 (0.95-1.13)
- 6	1.22 (1.10.-1.34)
- 7	1.36 (1.21-1.53)
- 8	1.27 (1.05-1.52)
- 9	1.46 (1.11-1.89)
- 10	2.57 (1.73-3.65)
- 11	1.58 (0.94-2.46)
- 12	0.53 (0.03-2.33)
- 13	5.13 (3.54-7.16)

- 14<br>- 15<br>- 16<br>- 17	-<br>0.64 (0.04-2.81)<br>-<br>5.19 (2.36-9.77)
**Heart failure**	1.25 (1.21-1.30)
**Renal failure**	1.33 (1.27-1.40)
**Cardiovascular risk burden**<br>- 0<br>- 1<br>- 2<br>- 3<br>- 4<br>- 5<br>- 6<br>- 7<br>- 8	 1<br>0.94 (0.74-1.20)<br>0.92 (0.73-1.17)<br>0.84 (0.67-1.07)<br>0.79 (0.63-1.01)<br>0.84 (0.67-1.07)<br>0.77 (0.61-0.98)<br>0.82 (0.64-1.07)<br>0.53 (0.13-1.42)
Values are displayed as odds ratios and 95% confidence intervals. Charlson Comorbidity Index (CCI); left atrial appendage closure (LAAC).	

Table S5. Results for hospital length of stay in patient subgroups stratified by patient comorbidity and sex

**Characteristics**	**CCI >3 (N=2,511)**	**CCI ≤3 (N=8,729)**	**Male sex (N=6,534)**	**Female sex (N=4,706)**
**Year of LAAC**				
- 2016	1	1	1	1
- 2017	0.90 (0.80-1.01)	0.96 (0.90-1.02)	0.98 (0.91-1.06)	0.89 (0.81-0.97)
- 2018	0.82 (0.74-0.92)	0.89 (0.84-0.95)	0.86 (0.80-0.93)	0.87 (0.80-0.94)
- 2019	0.81 (0.73-0.91)	0.84 (0.79-0.89)	0.86 (0.80-0.93)	0.78 (0.72-0.84)
**Sex**				
- Male	1	1		
- Female	1.30 (1.22-1.38)	1.08 (1.04-1.12)		
**CCI**				
- 0			1	1
- 1			1.03 (0.96-1.11)	1.10 (1.03-1.19)
- 2			1.10 (1.02-1.19)	1.12 (1.03-1.22)
- 3			1.13 (1.03-1.23)	1.21 (1.10-1.34)
- 4			0.96 (0.87-1.06)	1.12 (0.99-1.26)
- 5			0.94 (0.84-1.06)	1.17 (1.02-1.34)
- 6			1.10 (0.96-1.25)	1.29 (1.11-1.50)
- 7			1.06 (0.90-1.25)	1.76 (1.78-2.09)
- 8			1.01 (0.79-1.27)	1.77 (1.31-2.36)
- 9			0.98 (0.63-1.46)	1.94 (1.35-2.70)
- 10			1.93 (0.96-3.41)	3.04 (1.84-4.71)
- 11			0.86 (0.37-1.68)	3.44 (1.71-6.07)
- 12			-	0.55 (0.03-2.43)
- 13			2.30(0.98-4.49)	7.58 (4.93-11.2)
- 14			-	-
- 15			0.59 (0.03- 2.61)	-
- 16			-	-
- 17			5.17 (2.33-9.79)	-

Values are displayed as odds ratios and 95% confidence intervals. Each subgroup analysis was adjusted for the other 9 confounders of the primary model. Displayed are only confounders: year of LAAC, sex and CCI. Charlson Comorbidity Index (CCI); left atrial appendage closure (LAAC).

Table S6. Results for adverse discharge disposition in patient subgroups stratified by patient comorbidity and sex

Characteristics	CCI >3 (N= 2,511)	CCI ≤3 (N= 8,729)	Male sex (N=6,534)	Female sex (N=4,706)
**Year of LAAC**				
- 2016	1	1	1	1
- 2017	0.57 (0.27-1.19)	0.82 (0.50-1.37)	0.48 (0.26-0.89)	0.97 (0.55-1.79)
- 2018	0.42 (0.21-0.73)	0.80 (0.51-1.29)	0.49 (0.28-0.85)	0.79 (0.46-1.42)
- 2019	0.60 (0.32-1.17)	0.60 (0.38-0.96)	0.45 (0.27-0.77)	0.74 (0.44-1.31)
**Sex**				
- male	1	1		
- female	2.03 (1.35-2.81)	1.96 (1.48-2.63)		
**CCI**				
- 0			1	1
- 1			1.28 (0.65-2.60)	2.03 (1.15-3.71)
- 2			1.94 (0.97-3.98)	3.06 (1.67-5.73)
- 3			2.61 (1.21-5.71)	4.05 (2.02-8.21)
- 4			3.36, (1.45- 7.81)	2.96 (1.27-6.75)
- 5			2.62 (0.94-7.03)	3.78 (1.45-9.53)
- 6			2.86 (0.87-8.74)	7.99 (3.08-20.4)
- 7			8.32 (2.58-25.8)	8.05 (2.53-23.6)
- 8			2.72 (0.14-16.2)	18.7 (4.13-72.0)
- 9			6.97 (0.35-44.5)	18.6 (2.53-89.6)
- 10			-	-
- 11			-	-
- 12			-	-
- 13			183 (6.53-5159)	55.8 (2.01-1551)
- 14			-	-
- 15			-	-
- 16			-	-
- 17			-	-

Values are displayed as odds ratios and 95% confidence intervals. Each subgroup analysis was adjusted for the other 9 confounders of the primary model. Displayed are only confounders: year of LAAC, sex and CCI. Charlson Comorbidity Index (CCI); left atrial appendage closure (LAAC).

Table S7. Characteristics of dead patients stratified by sex		
**Characteristics**	**Male (N=6)**	**Female (N=10)**
**Year of LAAC**		
- 2016	1 (16.7%)	2 (20.0%)
- 2017	1 (16.7%)	2 (20.0%)
- 2018	2 (33.3%)	1 (10.0%)
- 2019	2 (33.3%)	5 (50.0%)
**Age (years)**	81 [75.5-85.25]	77 [71.0-76.05]
- Young and middle-aged (18-64 years)	0 (0%)	1 (10.0%)
- Senior (65-74 years)	3 (50.0%)	9 (90.0%)
- Gerontologic (≥75 years)	3 (50.0%)	6,822 (60.8)
**Ethnicity**		
- Caucasian	5 (83.3%)	9 (90.0%)
- Latin-American	0 (0%)	0 (0%)
- Afro-American	0 (0%)	0 (0%)
- Asian	1 (16.7%)	0 (0%)
- Native Americans	0 (0%)	1 (10.0%)
- Other	0 (0%)	0 (0%)
**Hospital size**		
- Large (≥250 beds)	5 (83.3%)	8 (80.0%)
- Medium (250-449 beds)	0 (0%)	2 (20.0%)
- Small (1-249 beds)	1 (16.7%)	0 (0%)
**Insurance**		
- Public	6 (100%)	10 (100%)
- Private	0 (0%)	0 (0%)
- Self-payment	0 (0%)	0 (0%)
- Other	0 (0%)	0 (0%)
**CCI**	4.00 [3.25-5.50]	3.00 [2.25-4.75]
- CCI >3	4 (66.7%)	4 (40.0%)
- CCI ≤3	2 (33.3%)	6 (60.0%)
**Heart failure**	3 (50.0%)	7 (70.0%)
**Renal failure**	5 (83.3%)	4 (40.0%)
**Cardiovascular risk burden**	3 [2.25-4.50]	3 [1.25-4]
**Hospital region**		
- New England	0 (0%)	1 (10.0%)
- Middle Atlantic	1 (16.7%)	2 (20.0%)
- East North Central	0 (0%)	0 (0%)
- West North Central	1 (16.7%)	0 (0%)
- South Atlantic	1 (16.7%)	4 (40.0%)
- East South Central	1 (16.7%)	0 (0%)
- West South Central	1 (16.7%)	0 (0%)
- Mountain	0 (0%)	2 (20.0%)
- Pacific	1 (16.7%)	1 (10.0%)
**CHADsVASc score**	3 [3-3.75]	5 [4-5.75]
**Simplified HAS-BLED score**	3 [3-4]	2.50 [1-3]

**Hospital location and teaching status**		
- Rural	0 (0%)	1 (10.0%)
- Urban and non-teaching	0 (0%)	1 (10.0%)
- Urban and teaching	6 (100%)	8 (80.0%)
Values are displayed as frequency (percent), mean (standard deviation) or median [interquartile range]. Charlson Comorbidity Index (CCI); left atrial appendage closure (LAAC).		
